# Beyond plaque segmentation: a combined radiomics-deep learning approach for automated CAD-RADS classification

**DOI:** 10.3389/fmed.2025.1536239

**Published:** 2025-03-26

**Authors:** Francesca Lo Iacono, Francesca Ronchetti, Anna Corti, Mattia Chiesa, Gianluca Pontone, Gualtiero I. Colombo, Valentina D. A. Corino

**Affiliations:** ^1^Department of Electronics, Information and Bioengineering, Politecnico di Milano, Milan, Italy; ^2^Department of Perioperative Cardiology and Cardiovascular Imaging, Centro Cardiologico Monzino IRCCS, Milan, Italy; ^3^Bioinformatics and Artificial Intelligence Facility, Centro Cardiologico Monzino IRCCS, Milan, Italy; ^4^Department of Biomedical, Surgical and Dental Sciences, University of Milan, Milan, Italy; ^5^Unit of Immunology and Functional Genomics, Centro Cardiologico Monzino IRCCS, Milan, Italy

**Keywords:** autoencoder, CAD patients, coronary computed tomography angiography, multiplanar reconstruction image, radiomics

## Abstract

**Introduction:**

Coronary Artery Disease (CAD) is a leading cause of global mortality, accurate stenosis grading is crucial for treatment planning, it currently requires time-consuming manual assessment and suffers from interobserver variability. Few deep learning methods have been proposed for automated scoring, but none have explored combining radiomic and autoencoder (AE)-based features. This study develops a machine learning approach combining radiomic and AE-based features for stenosis grade evaluation from multiplanar reconstructed images (MPR) cardiac computed tomography (CCTA) images.

**Methods:**

The dataset comprised 2,548 CCTA-derived MPR images from 220 patients, classified as no-CAD, non-obstructive CAD or obstructive CAD. Sixty-four AE-based and 465 2D radiomic features, were processed separately or combined. The dataset was split into training (85%) and test (15%) sets. Relevant features were selected and input to a random forest classifier. A cascade pipeline stratified the three classes via two sub-tasks: (a) no CAD vs. CAD, and (b) nonobstructive vs. obstructive CAD.

**Results:**

The AE-based model identified 17 and 6 features as relevant for the sub-task (a) and (b), respectively, while 44 and 30 features were selected in the radiomic model. The two models reached an overall balanced accuracy of 0.68 and 0.82 on the test set, respectively. Fifteen and 35 features were indeed selected in the combined model which outperformed the single ones achieving on the test set an overall balanced accuracy, sensitivity and specificity of 0.91, 0.91, and 0.94, respectively.

**Conclusion:**

Integration of radiomics and deep learning shows promising results for stenosis assessment in CAD patients.

## 1 Introduction

Coronary Artery Disease (CAD) represents one of the leading causes of morbidity and mortality worldwide, accounting for 8.9 million annual deaths (1). Coronary plaque progression may determine coronary lumen stenosis and impairing blood supply to the myocardium, thus potentially causing major adverse cardiac events (2). Grading coronary artery stenosis is a crucial step in the diagnosis of patients with suspected CAD, enabling tailored therapeutic interventions and clinical decision-making. Based on the Coronary Artery Disease-Reporting and Data System (CAD-RADS) (3), the standard coronary computed tomography angiography (CCTA)-based stenosis severity scoring, six distinct categories are defined: class 0 (no stenosis: 0%), class 1 (minimal nonobstructive stenosis: 1–24%), class 2 (mild nonobstructive stenosis: 25–49%), class 3 (moderate stenosis: 50–69%), class 4 (severe stenosis: 70–99%), and class 5 (total occlusion: 100%). However, the therapeutic approach is primarily guided by the degree of coronary stenosis, enabling a clinically relevant stratification into three fundamental classes: absence of stenosis (CAD-RADS 0), nonobstructive (CAD-RADS 1–2, stenosis<50%) and obstructive (CAD-RADS 3–4-5, stenosis>50%) stenosis.

It should be emphasized that CAD-RADS scoring requires a high-level of expertise from radiologists and it is subjected to interobserver variability, as well as high time-consumption (4). Furthermore, plaque segmentation represents a critical challenge frequently addressed in literature, especially in radiomics-based research (5–13). This is usually manually performed with open-source software (6, 7), requiring additional time and clinical staff, or it relies on semi-automated segmentation methodologies based on commercial software (8–11, 13). Some works also tested fully automatic deep learning (DL) approaches to achieve stenosis detection and segmentation, showing promising results (Dice coefficients of 0.83–0.94). However, developing a classification approach based on features extracted directly from the images would remove the segmentation issue, enhancing both robustness and reproducibility of the analysis and overcoming time-consumption problems.

With respect to stenosis grading, current literature reports a few radiomic-based studies (12–14). In these works different data representations, such as 2D patches (12), 3D plaque (13), or MPR CCTA images (15), and different classification schemes were explored. In Jin et al. (12) the authors focused on a five-classes (minimal, mild, moderate, severe and occluded) stenosis severity differentiation achieving an accuracy of 0.84, while in (13) and (14) a plaque-based binary classification into functionally significant or non-significant, was achieved with an accuracy of 0.74 and 0.92, respectively.

The DL literature presents a broader spectrum of methodological approaches, with studies employing different architectures with a strong focus on convolutional neural networks (CNN) (8, 15–22), along with Vision Transformers (20), transfer learning strategies (15, 16, 19), ConvLSTM architecture (8) and commercial tools (23). Conventional or MPR CCTA images were primarily used as input in these works, building end-to-end models for CAD-RADS classification. Different classification tasks were addressed with the binary one being the most common approach. DL studies have focused on the discrimination between two groups of CAD-RADS score, obtained with different stenosis threshold - mainly 50%, (0–1-2 vs. 3–4-5) (16, 18–20, 23–25) - achieving the highest performance with accuracy values from 0.60 to 0.99. Other studies addressed more challenging tasks achieving multi-class stratification (8, 15, 21, 22, 25, 26). Few of them (21, 22, 26) focused on stratifying the three CAD-RADS classes (0 vs. 1–2 vs. 3–4-5) providing valuable clinical information for therapeutic decision-making, with reported accuracy values ranging from 0.81 to 0.86.

Among the existing literature, two significant research gaps are found. First, no studies have explored the application of autoencoders (AEs) for stenosis evaluation. AEs encode the input data into a lower-dimensional space through an unsupervised learning approach, capturing essential data patterns independently of class labels. This characteristic provides greater flexibility with respect to other DL-architectures a CNN networks, as the extracted features can be applied to various downstream analyses beyond classification, including risk prediction or survival analysis. Second, combination of radiomic and DL-based features remains largely unexplored in the cardiac field, where only two existing studies focused on scar identification in hypertrophic patients (27, 28).

AEs, which compress input data into latent-space vectors through unsupervised learning, have not yet been explored for coronary artery MPR image reconstruction. Additionally, no previous research has investigated the integration of radiomic and AE-based features for stenosis evaluation. The advantage of this approach lies in the latent-space vector that contains a minimal representation of the input and it can be used as feature matrix, input to a machine learning model (29).

The aim of the present study is twofold: first to assess whether a simple AE model can be used to effectively compress MPR images. The second aim is to develop a machine learning pipeline assessing the value of radiomic features and AE-based features, singularly and in combination, for the automated patient-based evaluation of stenosis from CCTA.

## 2 Materials and methods

### 2.1 Patients and image dataset

The study population included 220 patients who underwent CCTA at IRCCS Centro Cardiologico Monzino (Milan, Italy) between 2016 and 2018 for suspected CAD. Clinical characteristics are shown in Table 1. The study was reviewed and approved by the institutional review board (registration number: R1061/19-CCM 11 25). All patients provided their written informed consent to participate in this study.

**Table 1:** Baseline characteristics of study population.

	All	No-CAD	Nonobstructive CAD	Obstructive CAD
n	220	40	80	100
Age (y), mean ± std	60.8 ± 12.0	51.4 ± 12.5	59.7 ± 11.2	65.6 ± 9.7
Male, n (%)	155 (70)	19 (48)	58 (73)	78 (78)
Hypertension, n (%)	77 (35)	8 (20)	28 (35)	41 (41)
Hyperlipidemia, n (%)	69 (31)	7 (18)	16 (20)	46 (46)
Diabetes, n (%)	19 (8)	0 (0)	2 (3)	17 (17)
Smoker, n (%)	40 (18)	5 (13)	11 (14)	24 (24)
Family history, n (%)	78 (35)	14 (35)	31 (39)	33 (33)

CCTA scans were acquired using Discovery CT 750 HD or Revolution CT (GE Healthcare, Milwaukee, IL) following the Society of Cardiovascular Computed Tomography guidelines (30).

CCTA images were evaluated by pairs of 10 experienced cardiac imagers (radiologists and cardiologists with 5–10 years of experience). Each scan was assigned a CAD-RADS score, with disagreements resolved by a senior cardiac imager with 10 years of experience. Each patient was assigned to one of these three classes: no CAD (0% stenosis, *n* = 40), nonobstructive CAD (stenosis<50%, *n* = 80) and obstructive CAD (stenosis≥50%, *n* = 100). The dataset is composed of CCTA longitudinal sections of the three main coronary arteries: left anterior descending, left circumflex, and right coronary artery. From the CCTA, MPR images were obtained by rotating images 45 degrees around the vessel centerline and subsequently analyzed. MPR images were derived for each of the three coronary artery, allowing visualization of the entire course of the vessel in 2D. Finally, the study considered a total of 340 coronary artery segments. As not all patients had all the eight MPR images, due to technical artifacts, a total of 2,548 MPR images was available for the analysis.

### 2.2 Region of interest definition

**Figure 1:**
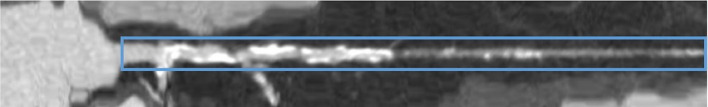
Example of multiplanar reconstruction image, i.e., a straightened coronary artery CCTA image, with superimposed the region of interest (blue solid line).

**Figure 2:**
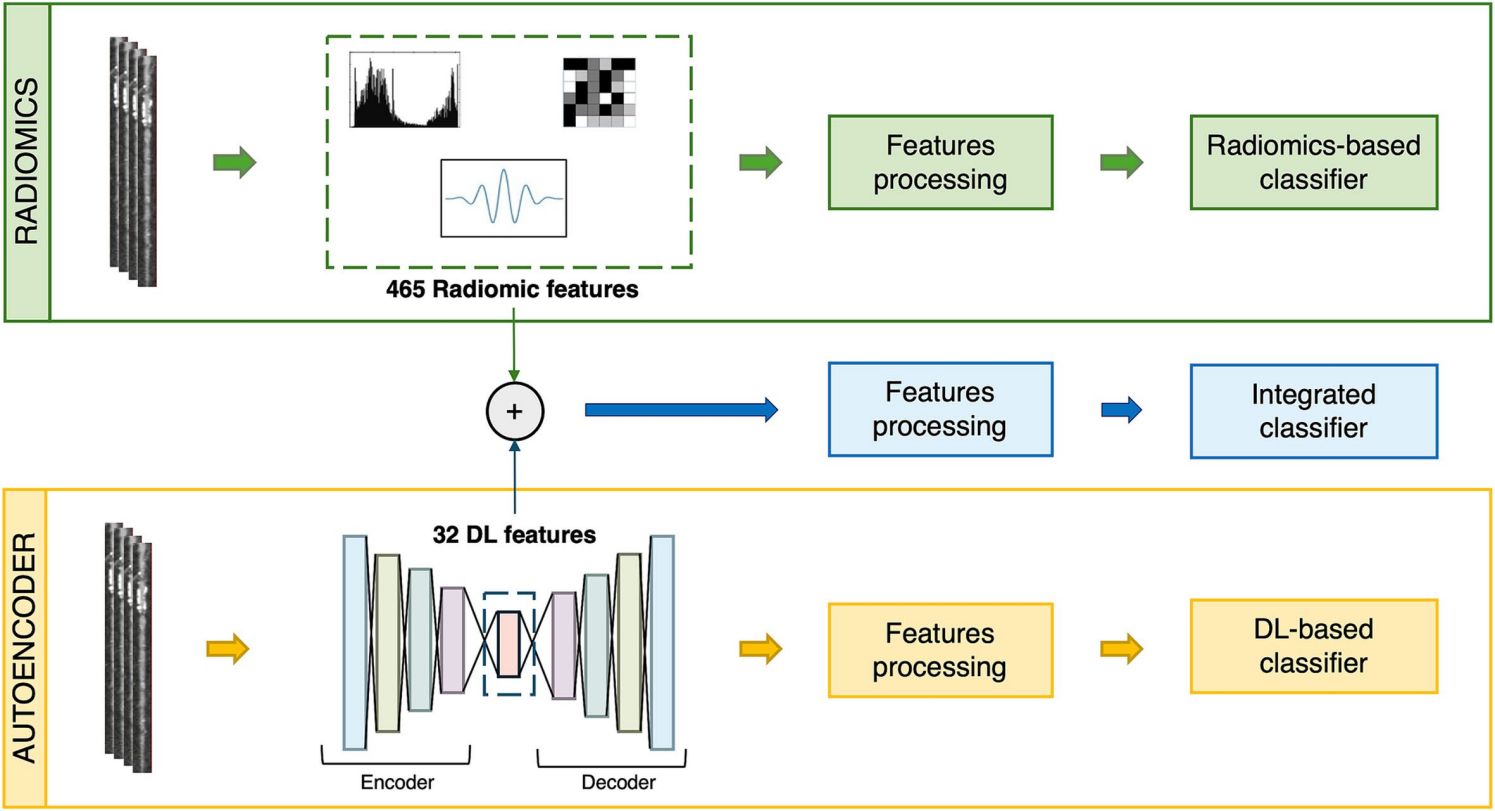
General workflow for radiomic, autoencoder (AE) and combined approach.

The region of interest (ROI) was defined as a 40-pixel wide rectangle, centered along the straightened coronary centerline. The ROI lower boundary aligned with the inferior margin of the image, while the upper boundary was defined to exclude the ventricular muscle (see Figure 1). ROI delineation was performed on a single view of a patient’s coronary artery and then applied to all the images of the same vessel. The images containing the ROIs constituted the input to both radiomics and DL analysis (see Figure 2 for the workflow).

### 2.3 Radiomic features extraction

Four-hundred sixty-five 2D radiomic features were extracted from both the original and filtered version of the images (Wavelet transformation) using Pyradiomics 3.0 (31). Default settings were considered for extracting the radiomic features, namely B-spline interpolation, and fixed-bin histogram discretization, with 25 bins. Four Wavelet decomposition images as HH, LL, LH, HL (where “H” stands for a high-pass filter and “L” for a low-pass filter) were considered. Thus, 18 first order statistics and 75 textural features (24 gray level co-occurrence matrix, 16 gray level run length matrix, 16 gray level size zone matrix, 5 neighboring gray tone difference matrix and 14 gray level dependence matrix) were computed from the original image and the 4 wavelet transforms, resulting in 465 radiomic features.

### 2.4 AE-based features extraction

AEs are unsupervised neural networks trained to reconstruct the input data by minimizing the error between the input and predicted output. An AE consists of three main components: the encoder, the decoder, and the latent-space representation. The encoder projects the input into a low-dimensional space, called latent space or bottleneck vector, while the decoder up-scales the latent space back to the original dimension. The decoder layers mirrored the encoder ones. As a product, AEs automatically extract a set of numerical features incapsulating the most important information.

In this study, three AE architectures were evaluated, considering different number of hidden layers and latent space vector size (256, 128, 64 neurons), to investigate the impact on prediction of different level of abstraction. The first AE (AE256) follows a design with one neuron layer of size 512 in the encoder, a latent space vector of size 256 and a symmetric decoder. The second architecture (AE128) introduces an additional layer of 256 neurons in both encoder and decoder and reduces the latent space to 128 neurons following a geometric progression in layer dimensions with a reduction factor of two (512 → 256 → 128). The third architecture (AE64) further extends this progression, introducing another layer of 128 neurons in the encoder and decoder (512 → 256 → 128 → 64), achieving the highest compression ratio. Across all architectures, ReLU activation functions are used in the intermediate layers to prevent vanishing gradients and introduce beneficial sparsity in neural activations, while linear activation in the bottleneck preserves the full range of encoded information, and sigmoid activation in the output layer ensures normalized reconstructions in the range [0,1]. The progressive reduction in layer dimensions (compression ratio ≈ 0.5 between consecutive layers) enables the network to learn hierarchical features capturing increasingly complex and abstract features with greater depth (32).

The AEs were trained by applying the backpropagation strategy setting the mean squared error between the input data x and its reconstruction x̂ as loss function. Adam optimizer was used with a maximum number of training epochs set to 300 and a batch size equal to 48.

The structural similarity (SSIM) index was used as metric to measure the similarity between the original image and the reconstructed one (33). The SSIM index ranges between −1 and 1, where 1 indicates perfect similarity, 0 indicates no similarity, and −1 indicates perfect anti-correlation. Once chosen the autoencoder architecture an ablation study was conducted by modifying various model’s parameters, such as learning rate from 0.1 to 0.001, loss function and optimization algorithm optimizer, to determine their contribution to the overall autoencoder reconstruction performance.

### 2.5 Machine learning pipeline

The dataset was partitioned into training (85%) and test set (15%), using a patient and label-stratified split. During the training phase, further 30 training (80%) and validation (20%) stratified splits were applied to assess the pipeline robustness. In the training phase, scaling and feature selection, as well as data balancing were applied.

In particular, feature selection was performed in three steps assessing non-redundancy and feature significance. The first step consisted of a correlation-based feature selection, performed to ensure a set of features with low internal redundancy. When a pair of features had an absolute Spearman correlation coefficient above 0.95, only one of the two was kept. In particular, the one with lower mean correlation with all the other features was selected. In the second step, a Wilcoxon rank-sum test was performed on each feature to identify the ones significantly differentiating the compared groups (no CAD vs. any CAD or nonobstructive vs. obstructive CAD). Finally, the least absolute shrinkage and selection operator (LASSO) was used to select the final set of features.

Data balancing in the training set was obtained by using the Synthetic Minority Over-sampling TEchnique algorithm (34).

Finally, a random forest classifier was trained on the entire training set, and then applied on the test set.

A cascade machine learning pipeline was developed to achieve a 3-class stratification through two simpler sub-tasks: (a) no CAD vs. any CAD and, then, (b) nonobstructive vs. obstructive CAD. The classification has been performed for each image, and the final patient classification was based on the majority voting criterion.

The above-described pipeline was used to develop a single radiomic model, an AE-based model and the combined model. In this last approach radiomic and AE features were merged together and submitted to all the feature selection and processing steps previously described.

The predictive performance of the model was evaluated through the balanced accuracy, sensitivity, specificity, f1-score, and Area Under the Curve of the Receiving Operating Characteristic curve (AUC-ROC). For multiclass tasks, the macro averaging version of f1-score, AUC-ROC, sensitivity, and specificity was used.

Finally, to provide deeper insights into the relationship between encoded image information and clinical variables, statistical analyses were performed on the AE-based features selected by the combined model. Mann Whitney U test was used to compared feature distributions between patient subgroups stratified according to available clinical characteristics reported in Table 1 (sex, hypertension, smoking status, hyperlipidemia, diabetes, family history). Additionally, a correlation analysis, using Spearman coefficient, was performed to evaluate the relationship between the selected AE-based features and patients’ age.

## 3 Results

### 3.1 AE reconstruction

**Figure 3:**
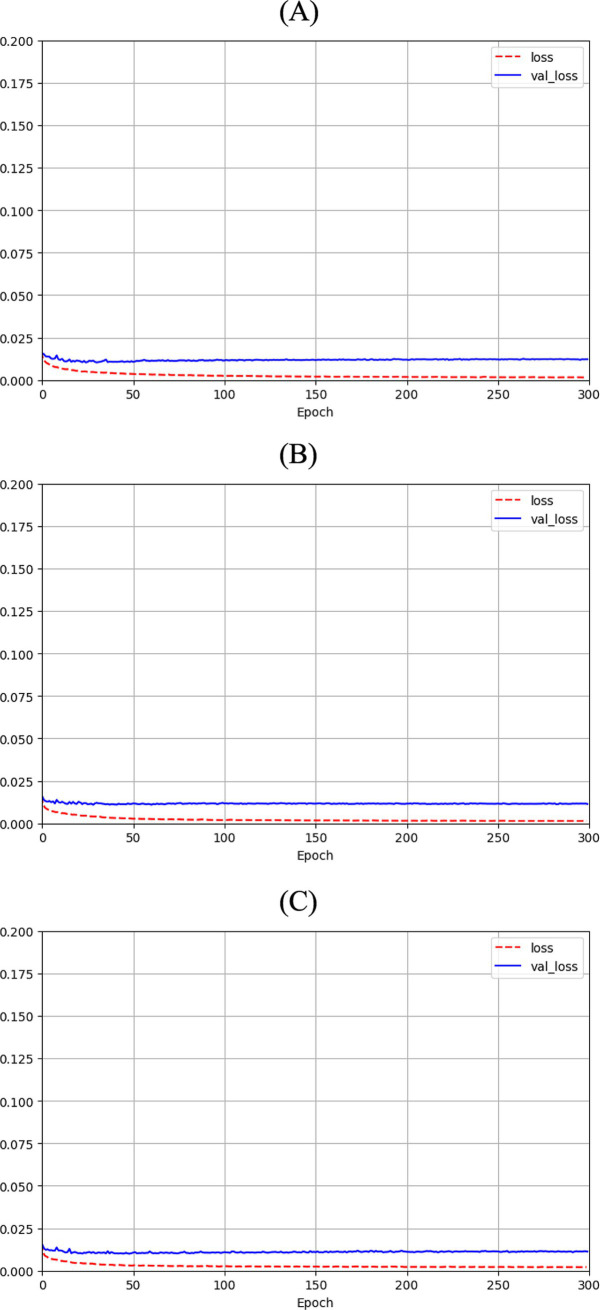
Autoencoder loss function with bottleneck size of **(A)** 64, **(B)** 128 and **(C)** 256 as function of epochs for training (dashed red line) and validation (solid blue line).

**Figure 4:**
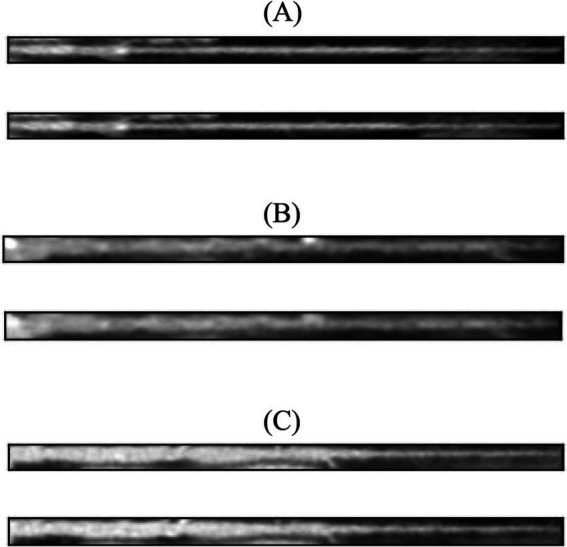
In each panel, the original image (top) is shown together with its reconstructed version (bottom) using AE64. **(A)** No CAD, **(B)** nonobstructive CAD, and **(C)** obstructive CAD. The SSIM index for these reconstructed images was 0.97, 0.96, 0.96, respectively. AE, autoencoder; SSIM, structural similarity.

**Figure 5:**
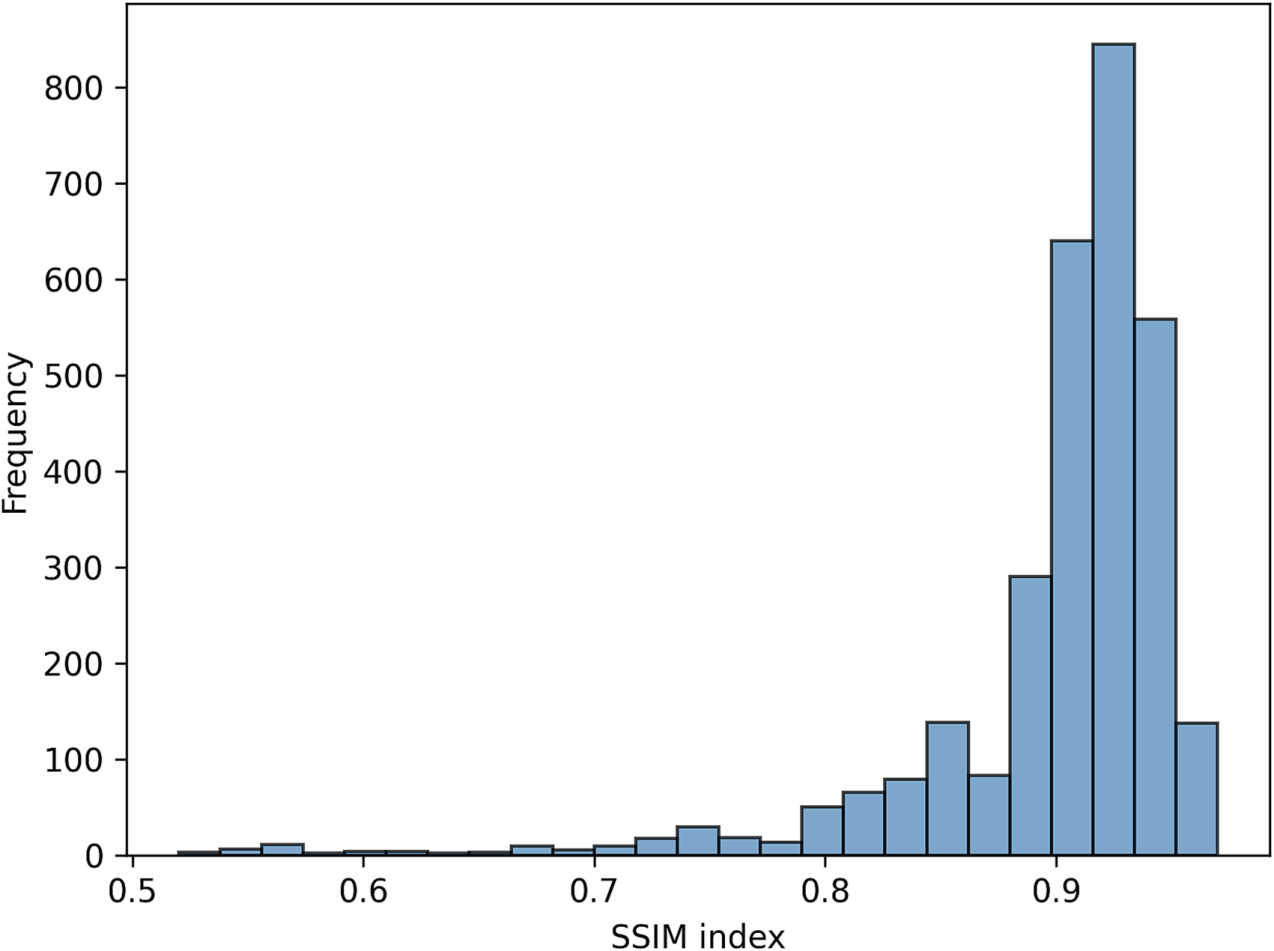
Distribution of the SSIM index for AE64 (autoencoder with latent space vector size = 64). AE, autoencoder; SSIM, structural similarity.

Figure 3 shows the loss function for the three AEs with the different latent space vector sizes: it can be noted that all three architectures present small values of the loss. The computational complexity for each of the three models, in terms of parameters number, training time and inference time, is reported in Table 2. It can be noticed that the differences among these metrics are negligible, with variations in the number of parameters less than 0.23% (41,152 parameters) between models, and comparable training and inference times. Moreover, the mean value of the SSIM index on the entire database was 0.90 ± 0.06, 0.90 ± 0.06, and 0.89 ± 0.07 for the AE with latent space vector size of 64, 128, and 256, respectively. Being the performance very similar, the most parsimonious model (i.e., the one with the smallest latent space vector size, named AE64) was chosen and used for further analysis. Supplementary Table S1 shows the AE64 performance across different model configuration. In Figure 4 can be seen an example of original and reconstructed images for the different classes, i.e., no CAD, nonobstructive CAD, and obstructive CAD. It is worth noting that in all three cases original and reconstructed images are very similar with SSIM index ≥0.96. Figure 5 shows the distribution of the SSIM index for AE64 over all the analyzed images. It can be observed that most images have a SSIM index higher than 0.8, with only 5% of the images reconstructed with a lower similarity.

**Table 2:** Analysis of computational complexity for the three autoencoder architectures, in terms of parameters number, training time and interference time.

Autoencoder architecture	Parameters (n)	Training time (s)	Mean inference time/image (ms)
AE64	18,100,160	2603.69	0.4 ± 0.016
AE128	18,091,904	2554.38	0.4 ± 0.080
AE256	18,059,008	5921.62	0.4 ± 0.045

### 3.2 Model building and validation

**Figure 6:**
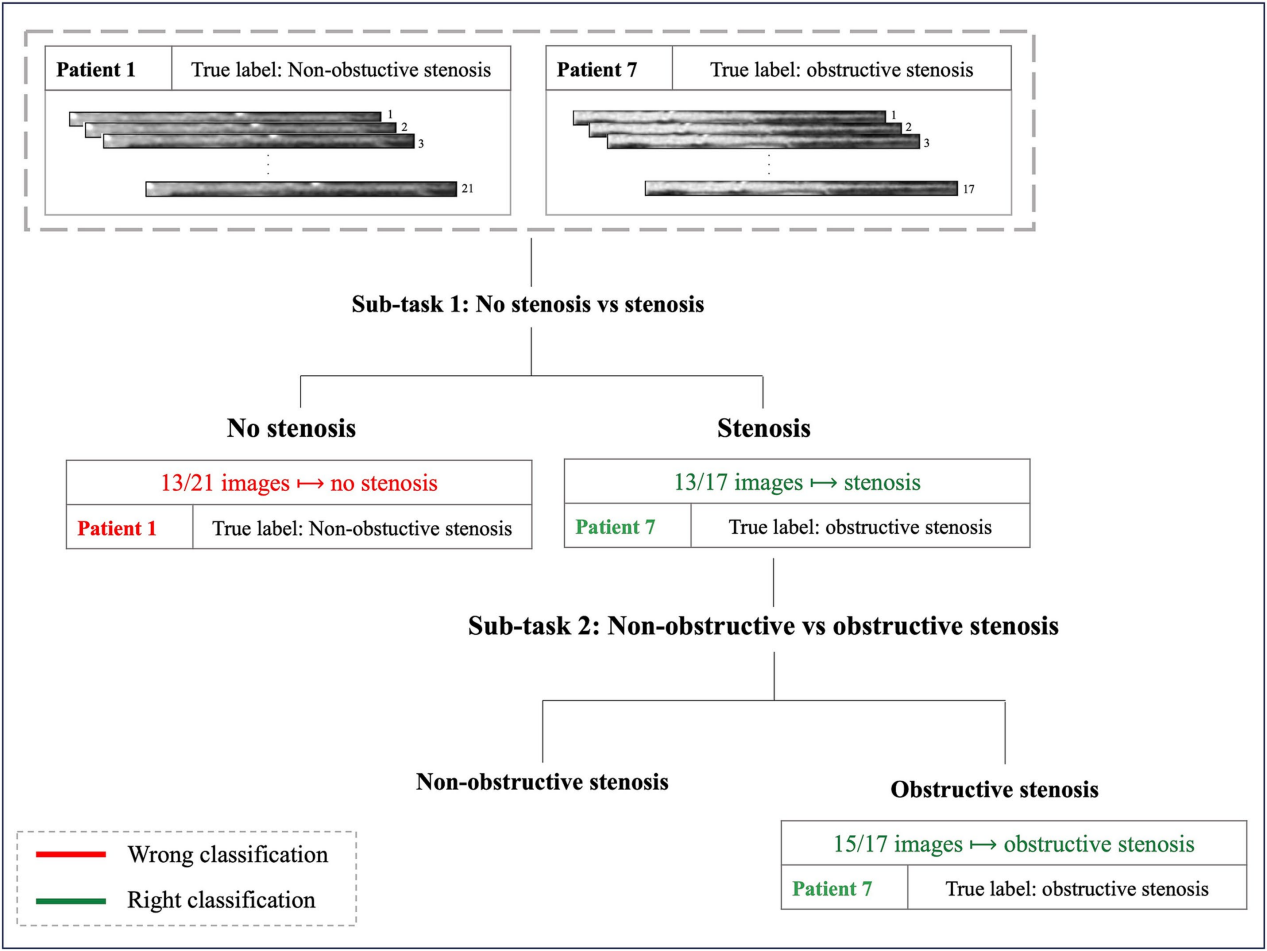
Examples of two patients’ classification. Patient 1, with 21 MPR CCTA images and a true diagnosis of non-obstructive stenosis, exemplifies a misclassification in the first sub-task: 13 out of 21 images were classified as no-stenosis, preventing progression to the subsequent classification step. Patient 7, with 17 images and a true diagnosis of obstructive stenosis, represents an example of successful classification: 13 out of 17 images were correctly identified as stenosis in the first step, allowing progression to the final sub-task where the patient received the correct classification (obstructive stenosis).

Figure 6 illustrates the cascade system employed to determine the final patient’s label reporting two examples of classification.

**Figure 7:**
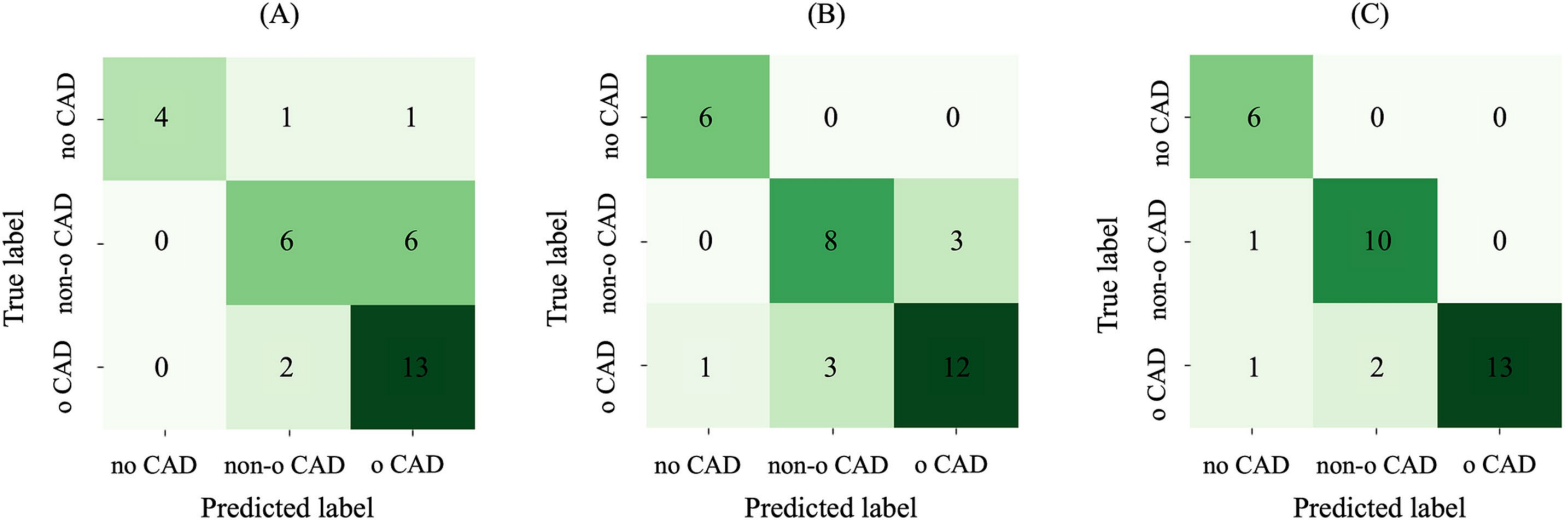
Confusion matrix on the test set using **(A)** AE-based features, **(B)** radiomic features and **(C)** their combination. AE, autoencoder; Non-o, nonobstructive; o, obstructive; CAD, coronary artery disease.

When considering AE-based features, i.e., features extracted from the bottleneck layer of the AE64, the feature selection step identified 17 and 6 features for the sub-task (a) and (b), respectively. Table 3 shows the performance metrics on the training-validation splits for the AE64. It can be observed that sub-task (a) obtained better performances than that of sub-task (b), with significant statistical differences (*p* < 0.001) for all the metrics except sensitivity. Figure 7A shows the confusion matrix on the test set, associated with the following metrics: balanced accuracy = 0.68, macro sensitivity = 0.68, macro specificity = 0.82 and macro AUC = 0.75.

**Table 3:** Performance metrics on 30 stratified training-validation splits.

Model	Metric	Task (a)	Task (b)
AE-based features	Balanced accuracy	0.72 ± 0.02	0.62 ± 0.02
	Macro sensitivity	0.79 ± 0.09	0.76 ± 0.09
	Macro specificity	0.72 ± 0.02	0.62 ± 0.02
	Macro F1-score	0.84 ± 0.05	0.69 ± 0.04
	Macro AUC	0.77 ± 0.06	0.62 ± 0.07
Radiomic features	Balanced accuracy	0.86 ± 0.02	0.71 ± 0.02
	Macro sensitivity	0.88 ± 0.05	0.75 ± 0.09
	Macro specificity	0.83 ± 0.04	0.72 ± 0.02
	Macro F1-score	0.90 ± 0.03	0.74 ± 0.04
	Macro AUC	0.90 ± 0.05	0.78 ± 0.05
AE-based and radiomic features	Balanced accuracy	0.88 ± 0.04	0.73 ± 0.03
	Macro sensitivity	0.88 ± 0.05	0.77 ± 0.07
	Macro specificity	0.88 ± 0.03	0.73 ± 0.03
	Macro F1-score	0.92 ± 0.02	0.76 ± 0.04
	Macro AUC	0.92 ± 0.03	0.79 ± 0.06

Values are expressed as mean ± standard deviation. AE, autoencoder.

When considering radiomic features, the feature selection step identified 44 and 30 features for the sub-task (a) and (b), respectively. Table 3 shows the performance metrics on the training-validation splits. Similar to the AE64, also radiomics obtained better performances on sub-task (a), with performance metrics significantly higher (*p* < 0.001) than that of sub-task (b). Figure 7B shows the confusion matrix on the test set, associated with the following metrics: balanced accuracy = 0.82, macro sensitivity = 0.82, macro specificity = 0.88 and macro AUC = 0.84. Comparing results in Figures 7A,B, it can be observed that differently from the AE-based model, the radiomic one correctly classified all the patients without CAD, and it also outperforms the prediction of patients with nonobstructive and obstructive CAD.

When combining radiomic and AE-based features, 50 and 35 features were selected for sub-task (a) and B, respectively. Most of the selected features were radiomic, with 8 AE-based features selected in the first sub-task and 6 in the second one. Comparing the two subtasks, sub-task (a) obtained significantly higher values (*p* < 0.001) in all the metrics score. Table 3 shows the performance metrics on the training-validation splits. Overall, the model combining AE-based and radiomic features provided better performance with respect to both AE64 and radiomic models, with statistically significant difference observed in balanced accuracy, f1 score and AUC.

Figure 7C shows the confusion matrix on the test set for the model based on combined radiomic and AE-based features. It can be observed that the prediction of both nonobstructive and obstructive CAD was improved, with a total of only 4 wrong classifications, compared to 10 and 7 wrong classifications for AE and radiomic-based model, respectively. The performance metrics for the combined model were: balanced accuracy = 0.91, macro sensitivity = 0.91, macro specificity = 0.94 and macro AUC = 0.89.

The results of statistical analyses examining the relationship between the 6 AE-based features selected by the combined model and clinical variables are reported in Supplementary Figures S1, S2. All the AE features exhibit at least two significant differences between patient subgroups stratified by the clinical characteristics. Feature 20 stands out by showing statistical significance across nearly all clinical variables (except hyperlipidemia), suggesting its ability to capture a comprehensive cardiovascular risk profile. Other features demonstrate more targeted findings: Features 57 and 58 show significant differences in subgroups defined by hypertension, sex, and smoking status, while features 27, 31, and 62 exhibit differences specifically in groups characterized by hypertension and smoking status. The Spearman correlation analysis with age, instead, revealed weak associations, with correlation coefficients not exceeding 0.17 in absolute value.

## 4 Discussion

Automated evaluation of stenosis from CCTA is an open challenge, and the detection and classification of coronary artery plaques are essential for CAD prevention and treatment. In this study, we proposed a novel approach based on the integration of features extracted by radiomics and by an AE, to perform a patient therapy-driven stratification into three classes: absence of stenosis (CAD-RADS 0), nonobstructive (CAD-RADS 1–2, stenosis<50%) and obstructive (CAD-RADS 3–4–5, stenosis>50%) stenosis. This task was addressed using CCTA-based MPR images. The main findings of the study are: (i) a simple AE with bottleneck size of 64 can efficiently reconstruct the MPR images of a coronary artery and (ii) the machine learning integration of radiomic and AE-based features improves the performance of single domain reaching a balanced accuracy of 0.91. The AE64 was able to reconstruct the MPR images with high similarity (average SSIM = 0.90), with only 5% of images with a SSIM index lower than 0.80. No significant improvement was found when a larger latent space vector size was considered (128 or 256 samples), highlighting the effectiveness of the proposed AE64.

The analysis was further supplemented by investigating potential relationships between AE-based features and patient clinical characteristics through statistical testing. The findings demonstrated that specific AE-based features revealed distinct patterns with particular patient subgroups defined by clinical variables such as hypertension, smoking status, and other cardiovascular risk factors. This suggests that the AE’s computational representation may reflect meaningful patient-level characteristics, in the context of automated CCTA analysis for patient stenosis scoring.

Current literature reports several studies addressing stenosis grading from CCTA images, which has become a crucial non-invasive imaging modality for comprehensive plaque evaluation (35, 36).

Several machine learning models using different input parameters, as healthy area of lumen estimation (37), vessel intensity and geometric features (38), multiple scales describing the properties of stenotic lesions (39), computational fractional flow reserve derived data (40), or radiomic features (12, 13), have been proposed.

Few studies (12–14) have explored the radiomic-based approach. Jin et al. (12) classified plaques into five classes (minimal, mild, moderate, severe and occluded), reaching an accuracy of 0.84 in the test set. However, their analysis was limited to 2D patches containing the lesion, performing a plaque-based analysis rather than adopting the clinically relevant patient-level stenosis classification approach used in the present study. Li et al. (13) focused on classifying plaques as functionally significant or non-significant according to the fractional flow reserve values (with 0.8 as cutoff), achieving an accuracy of 0.74 on the test set. Unlike the present study, which analyzed the full spectrum of cases from normal coronary arteries (0% stenosis) to severe stenosis (>50%), their study was limited to patients with at least one lesion stenosis degree between 30 and 90%, i.e., mild-to-severe stenosis. Notably, also this research employed a plaque-based classification instead of a patient-based assessment. While the current work outperformed both studies (balanced accuracy of 0.91), direct comparisons are challenging due to fundamental differences in the analyzed data (2D patches and 3D plaques versus MPR images) and classification objectives.

DL has gained increasing attention as a tool for coronary stenosis evaluation in CCTA. Different architectural solutions have been proposed in literature, varying both in their input data format and classification objectives. Several studies employed standard CCTA images. Jin et al. (16) applied a transfer learning-based ResNet18 model to classify coronary stenosis as normal (stenosis rate < 50%) or abnormal (stenosis rate > 50%). Although they reported a high accuracy of 0.99, their study was limited to a basic binary classification and a relatively small dataset of 126 images. A similar binary approach was developed in Han et al. (23), where a commercial CCTA-based artificial intelligence platform was employed for binary classification using either 50% or 70% stenosis as cutoff values, achieving AUCs of 0.85 and 0.78, respectively, on 318 patients. Conventional CCTA images were also used by Lin et al. (41) employing ConvLSTM network to address a different discrimination task into five CAD-RADS classes (1-2-3-4-5). While their multicenter study achieved a balanced accuracy of 0.87 on the test set, it required a substantially larger training dataset (921 patients, 5,045 lesions) compared to our method.

Other studies investigated different input data representations. Muscogiuri et al. (17) performed various classifications analyzing 121 CCTA MPR images per patient, placing them in an 11×11 squared image input to a CNN model. Which might lead to lose lesion details. When considering a three-class analysis (0 vs. 1-2 vs-3-4-5), they obtained an accuracy value of 0.60, which is lower than our results. Differently from the current study, their approach focused on classifying individual lesions rather than providing a comprehensive patient-level assessment, and the final results might have been overestimated since no clear patient-based splitting strategy between training and test sets was specified. Additionally, compression of images into an 11×11 format can lead to loosing crucial lesion details. In (18), the authors focused on different anatomical structures, i.e., left ventricular myocardium, extracting its characteristic features using a CNN-based architecture. Patients were classified according to the presence of functionally significant stenosis using a support vector machine classifier based on the left ventricle features, thus not providing analysis at the coronary vessel level.

Similar to our study, few researches (15, 19, 20, 24, 25) relied on MPR image stacks which allow to display the complete course of a vessel in 2D (25). CNN-based networks were largely employed among these works. In Tejero-de-Pablos et al. (19) a pre-trained CNN was used to extract texture features from MPR images of 57 patients getting an accuracy value of 0.80 on a leave-one-out cross-validation, to predict significant stenosis (obstruction >50%). This method was limited by both its small sample size and its focus on individual lesion classification rather than providing a comprehensive patient-level assessment, neglecting the overall coronary context in the decision-making process. A lesion-specific analysis approach is also presented in the study by Gupta et al., (24) that tested different DL models (EfficientNet, ResNet15, DenseNet16, Inception-ResNet) to detect significant stenosis in individual coronary arteries (left anterior descending artery (LAD), right coronary artery, or left circumflex artery), using two distinct stenosis thresholds: 50% (CAD-RADS 0-1-2 vs. 3-4-5) and 70% (CAD-RADS 0-1-2-3 vs. 4–5). Maximum AUC values of 0.95 and 0.94 was obtained using the LAD-based model using a 50 and 70% as thresholds, respectively. However, the study included a small number of positive cases (36% for the 50% threshold and 19% for the 70% threshold), which may limit the reliability of these findings. The same two cut-off values for binary classification were tested in Verpalen et al. (20) where pre-trained CNN architectures (21, 42) performed a patient-based CAD-RADS scoring analysis. Fifty patients and 148 vessels, were evaluated reaching a maximum accuracy value of 0.82 and 0.94 for the 50 and 70% stenosis thresholds, respectively. Similarly, study (15) employed a 2.5 CNN for patient-based classification, achieving an accuracy of 0.865 in binary stratification (0 vs. 1-2-3-4-5) and extending the analysis to a complete six-class CAD-RADS categorization (0–1–2-3-4-5) which got an accuracy of 0.825. A different multi-class analysis was performed by Penso et al. (25), who developed a token-mixer architecture to address both binary (50% stenosis threshold) and four-class CAD-RADS classification (0 vs. 1–2 vs. 3–4 vs. 5). Their method achieved an accuracy of 0.87 in the binary task but the performance decreased to 0.72 when extending the analysis to a four-class stratification. Notably, these studies reported performance values lower or comparable with respect to ones achieved in the current study. However, a direct comparison is challenging due to different analytical approaches from individual lesion evaluation to distinct CAD-RADS classification tasks.

Considering a three-class (0 vs. 1-2 vs. 3–4-5) stratification task using MPR images, literature reports few studies achieving it. Paul et al. (21) used an inceptionV3 neural network to obtain a patient-based classification. Their approach, based on majority voting of nine curved multiplanar reformatted CCTA images achieved a balanced accuracy of 0.81, lower than the performance obtained in the current study (accuracy of 0.91). Their study was limited by an unbalanced dataset with underrepresentation of CAD-RADS 3 cases (7.5% of patients) and a predominance of non-obstructive cases (71.6%). Moreover, the model showed a considerable number of false positives, with 15.1% of normal cases being misclassified as nonobstructive stenosis, potentially leading to unnecessary additional testing in clinical practice. Zreik et al. (22) implemented a multi-task recurrent CNN on a dataset of 163 patients (98 for training, 65 for testing), showing poorer performance compared to the current study with an accuracy of 0.75. A more recent study by Gerbasi et al. (26) explored a fine-tuned multi-axis Vision Transformer architecture for both binary (0-1-2 vs. 3-4-5) and three-class (0 vs. 1-2-3 vs. 4-5) categorization, reaching accuracy values of 0.82 and 0.86, respectively. While this approach eliminated the need for image annotations, the limited dataset size raised concerns about the robustness of the trained network.

It should be noted that most of the cited works employed DL architectures different from AEs whose choice was driven by its unique ability to perform unsupervised dimensionality reduction, compressing the input 2D images from 35,014 samples (854 × 41) to a compact 64-sample latent space vector while maintaining high reconstruction fidelity. This approach offers several advantages over different DL architectures such as CNNs: while CNNs are inherently supervised and specifically designed for classification tasks, AEs provide greater flexibility through their unsupervised learning framework, making them suitable for various downstream analyses beyond classification. In addition, all the reported studies employed single domain data, such as radiomics or DL. Only one previous investigation (14) explored the combination of these two domains, but with substantial methodological differences from the present study: their analysis was restricted to a binary classification task (high-degree stenosis >50% versus low-degree stenosis <50%), focused on coronary-level rather than patient-level assessment. Additionally, differently from the present analysis, the authors used only shape descriptors from segmented lesions as input to a CNN-based network, rather than integrating radiomic and DL features in a comprehensive analysis framework. Moreover, to the best of authors’ knowledge, in the cardiac imaging field, only two studies (27, 28) combined DL with radiomics, employing CNN-based architectures rather than AE. Compared to the literature, the present study introduced major novelties in the field: (i) it pioneered the use of autoencoder architecture for DL-based coronary feature extraction and integrated them with radiomic features extracted directly from MPR images and (ii) proposed a segmentation-free approach. As regards the first aspect, the developed approach provided a more comprehensive image characterization combining mathematically-defined radiomic features that quantify image characteristics (such as texture and intensity distribution), with learned compressed representations that could capture additional information. As regards the second aspect, a segmentation-free approach represents a major advancement, by overcoming the time-consumption issue and enhancing the robustness and reproducibility of the analysis while maintaining high performance (overall balanced accuracy of 0.91 on the test set).

Considering all the above, the present study proposed a novel strategy for coronary stenosis grading and achieved better diagnostic performance compared to the state-of-the-art models addressing patients’ therapy-driven stratification.

Several limitations should be acknowledged in this study. First, the dataset presented significant class imbalance, which could affect the model’s generalization capabilities. Second, the single-center nature of the data collection and the absence of external validation limited the assessment of the algorithm’s generalizability across different clinical settings. Ultimately, even if some relations between the AE features and clinical variables were found, an exhaustive interpretation of this AE-derived representation, along with established direct clinical correlations with patients’ medical characteristics, remained challenging.

Future developments will focus on addressing these limitations through multiple approaches. A crucial enhancement would involve expanding the dataset to include multiple centers, thereby increasing both sample size and population diversity to improve the model’s robustness. Additionally, testing the model on external validation sets, from different imaging datasets, would provide a rigorous assessment of the model’s generalization capabilities. Another interesting development would be the implementation of interpretability strategies to link bottleneck features to specific image regions, providing clinicians with more transparent and interpretable results. Such improvements would represent crucial steps toward potential clinical implementation of the proposed methodology.

## 5 Conclusion

In the current study, for the first time in literature, CCTA-based MPR images of coronary arteries were used to develop a machine learning model combining radiomic and AE-based features. In particular, a cascade pipeline stratified the three patient classes via two sub-tasks: no CAD vs. CAD, and nonobstructive vs. obstructive CAD. The combined approach showed evident improvements with respect to the single radiomic and AE-based models: a higher overall balanced accuracy was achieved and the prediction accuracy for both nonobstructive and obstructive CAD improved, with only 4 misclassifications, compared to 10 and 7 obtained with the AE-based and radiomics-based models, respectively. Also, it is important to highlight that these results were achieved using a simple AE having a bottleneck size of 64 able to reconstruct 95% of MPR images with an SSIM higher than 0.80. The automated patient stratification in the three stenosis grade classes (no stenosis, nonobstructive, and obstructive coronary stenosis) hold high clinical significance, particularly for therapeutic decision-making.

## Data Availability

The raw data supporting the conclusions of this article will be made available by the authors, on reasonable request. Requests to access these datasets should be directed to gianluca.pontone@cardiologicomonzino.it.
