# Supplementary material for: Beyond plaque segmentation: a combined radiomics-deep learning approach for automated CAD-RADS classification
Source: Front Med (Lausanne). 2025 Mar 26;12:1536239. doi: 10.3389/fmed.2025.1536239 (PMC11979263; doi:10.3389/fmed.2025.1536239)

Supplementary material

Table S1: Performance metrics of the autoencoder (bottleneck size 64) across different hyperparameter configuration. SSIM: structural similarity index; MSE: mean squared error; MAE: mean absolute error; PSNR: peak signal-to-noise ratio.

Configuration	Optimizer	Learning Rate	Loss Function	SSIM	MSE	MAE	PSNR
**Model 1**	Adam	0.001	MSE	0.9089	0.0035	0.0392	26.7182
**Model 2**	RMSProp	0.001	MSE	0.8580	0.0072	0.0610	22.1284
**Model 3**	SDG	0.001	MSE	0.5395	0.0455	0.1861	13.6542
**Model 4**	NAdam	0.001	MSE	0.9078	0.0040	0.0410	26.3639

Configuration	Learning Rate	Optimizer	Loss Function	SSIM	MSE	MAE	PSNR
**Model 5**	0.1	Adam	MSE	0.1689	0.1377	0.3422	8.6830
**Model 6**	0.01	Adam	MSE	0.1710	0.1342	0.3387	8.8021
**Model 7**	0.001	Adam	MSE	0.9089	0.0036	0.0393	26.7210
**Model 8**	0.0001	Adam	MSE	0.8971	0.0038	0.0425	25.5934

Configuration	Loss Function	Optimizer	Learning Rate	SSIM	MSE	MAE	PSNR
**Model 9**	MSE	Adam	0.001	0.9089	0.0035	0.0392	26.7182
**Model 10**	Binary cross entropy	Adam	0.001	0.8956	0.0036	0.0393	26.5531
**Model 11**	MAE	Adam	0.001	0.8853	0.0041	0.0396	25.6095

Figure S1: Statistical analyses (Mann Whitney U test) performed on autoencoder features to compare distributions between patient subgroups stratified according to clinical characteristics. The obtained p-values are reported in each heatmap cell with asterisks specificying the statistical significance of the analysis (* p<0.05, **p<0.01, ***p<0.001).

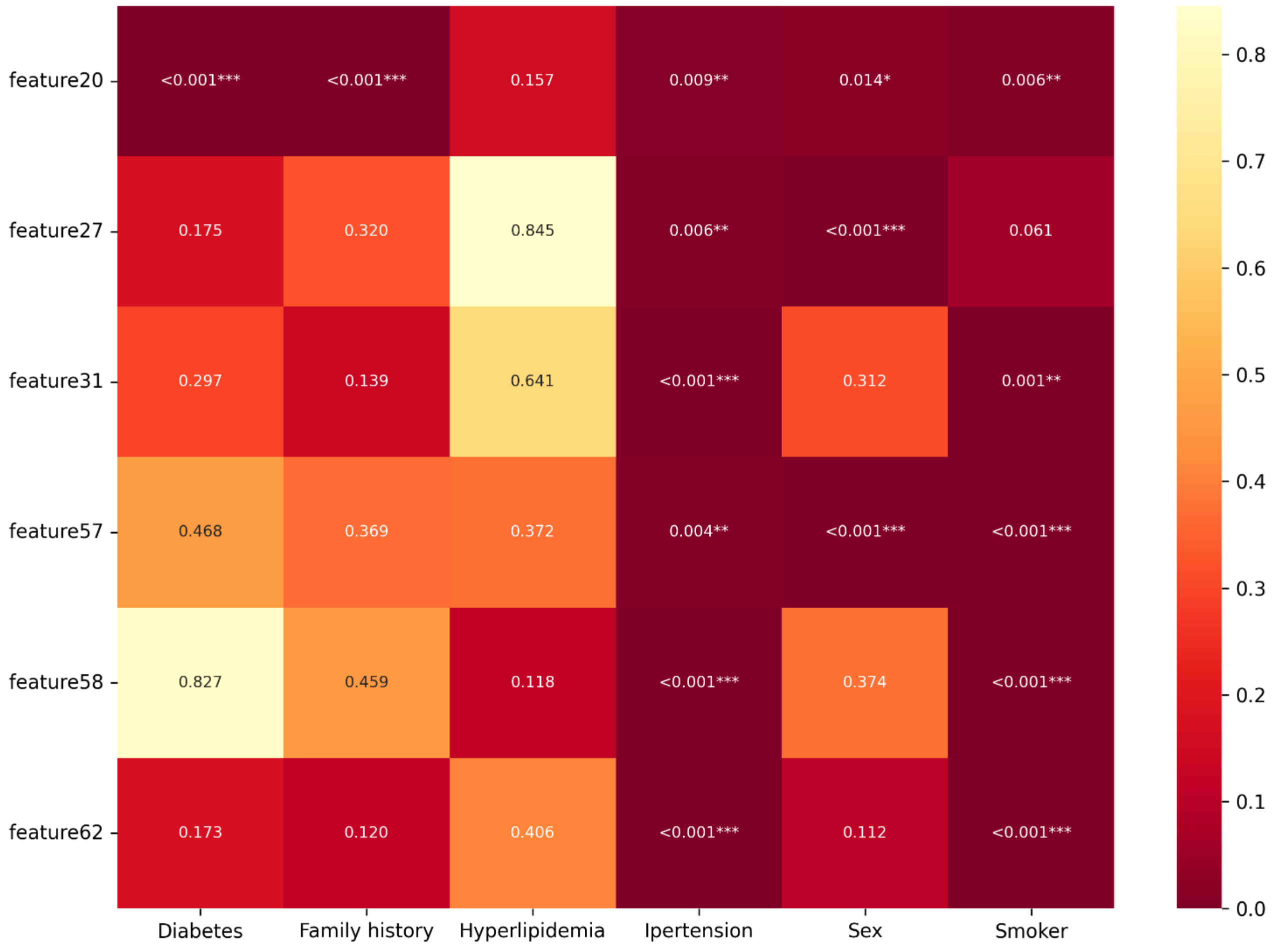

Figure S2: Spearman correlation analysis between autoencoder features and patients' Age variable. Asterisks specificy the statistical significance of the analysis with * p<0.05, **p<0.01, ***p<0.001.

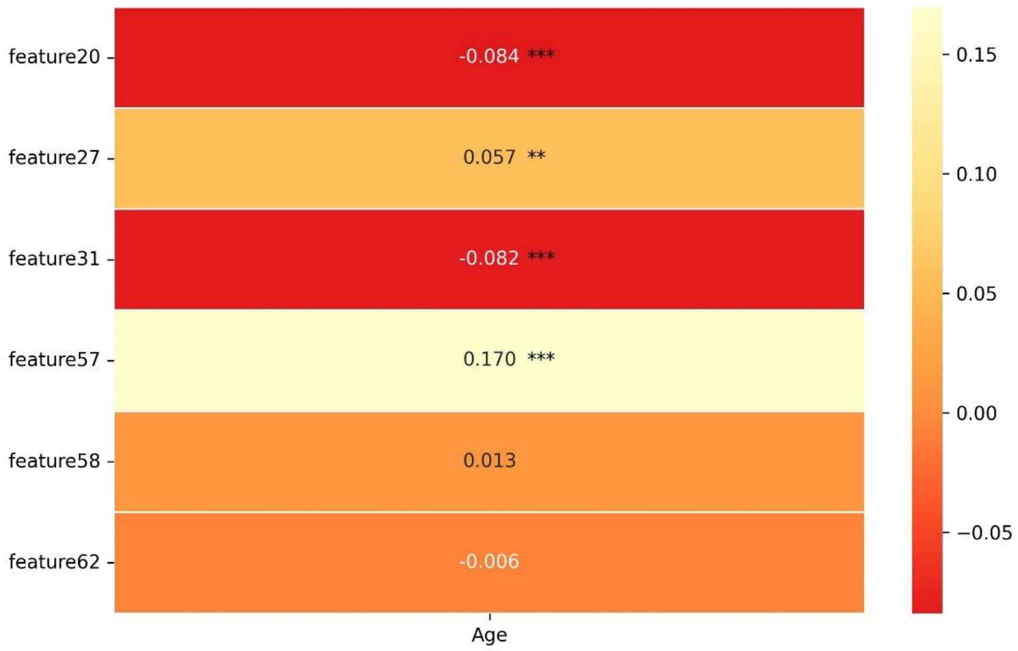

Supplement: Supplementary file 1 [file Data_Sheet_1.pdf]
